# A Secure User Interface for Preclinical Evaluation of AI in Patient Portal Message Management: Tutorial

**DOI:** 10.2196/83216

**Published:** 2026-07-20

**Authors:** Kelly Gleason, Thomas Kidu, Vignesh Babu, Brian Hasselfeld, Jennifer Wolff

**Affiliations:** 1 School of Nursing, Johns Hopkins University, 525 N. Wolfe Street, Baltimore, MD, 21215, United States, 1 708334876; 2 Whiting School of Engineering, Johns Hopkins University, Baltimore, MD, United States; 3 Bloomberg School of Public Health, Johns Hopkins University, Baltimore, MD, United States

**Keywords:** patient portal, management, electronic health records, artificial intelligence, AI, large language models, tutorial

## Abstract

The growing use of AI to support patient portal message management requires rigorous preclinical evaluation. Directly testing AI within electronic health record (EHR) systems poses significant safety, workflow, and data-governance risks. Here, we present a technical feasibility report on a secure user interface (UI) sandbox designed to enable clinical and technical teams to experiment with AI for portal messaging before clinical integration. In this context, a “sandbox” refers to a controlled, nonproduction environment that allows safe testing, prompt iteration, and evaluation of AI outputs without impacting live EHR systems or patient care. We developed a web UI in Python 3 with a modular backend for data handling and AI task execution that operates entirely within the institutional firewall. The system runs in a secure research environment equipped with an NVIDIA GRID T4-1Q graphics processing unit (GPU) and institutional access controls. We designed a deidentification pipeline to remove or replace personal health identifiers and assessed its precision. The platform supports single-message and batch workflows and exposes example large language model (LLM)–enabled tasks such as authorship identification, message categorization, criticality flagging, and response drafting using zero-shot, one-shot, and few-shot prompting. The system successfully executed end-to-end workflows to ingest messages, run individual or batch AI analyses, and present outputs for review. Personal health information partial masking was applied across the corpus using a deidentification pipeline validated against 110 manually adjudicated entities (sensitivity 95.1%, precision 82.1%). We ran use cases with an institutional review board–approved corpus of a dementia-relevant subset of 6941 patient portal messages categorized as “medical advice requests” from 497 unique patients. With the support of the UI, we tested which prompting strategies yielded interpretable outputs for authorship identification, categorization, and criticality flagging, and whether response drafting produced editable clinician starting points. A token-based cost readout provided transparent operating estimates for LLM-backed tasks. This framework offers a practical, secure path to test AI behavior on real messages without affecting live EHR workflows and thus supports exploratory testing, prompt iteration, and comparative analyses, including LLM prompts versus baseline models, while preserving governance boundaries. We discuss design choices, safety controls, and the limits of a sandbox approach. A secure, UI-based sandbox enables health system teams to evaluate AI for patient portal messaging before clinical integration. The goal is not to assume benefit but to generate evidence about feasibility, risks, and fit to clinical needs in a controlled setting.

## Introduction

Patient portal messaging has expanded rapidly and now encompasses clinical questions, symptom updates, and coordination tasks that can affect care quality and timeliness [1-4]. As message volumes rise, concerns about clinician workload and burnout have grown [2]. In parallel, health systems are exploring AI to help manage message flow through triage support, authorship cues, categorization, and draft responses [5]. Despite optimism that AI may help, its true impact on workload, safety, and equity remains uncertain [6-8].

The increasing use of AI to support patient portal message management requires rigorous testing and evaluation. Patient portal messages convey critical health information, from appointment reminders and laboratory results to educational content, making clarity, accuracy, and accessibility essential. Even minor ambiguities or technical errors in poorly designed AI can lead to misunderstandings and compromise care quality [9-11].

Developing AI tools for portal inbox management is particularly challenging within live electronic health record (EHR) systems. Direct integration at early stages can introduce significant risks, including threats to data integrity and barriers to agile development [12]. The sensitive nature of health information necessitates a secure, isolated environment to test messaging functions without endangering patient safety or system stability. A dedicated testing platform is therefore critical because it must realistically simulate message flows, support the creation and evaluation of dynamic content, and enable real-time interactions with patients and clinicians. Importantly, this environment should remain separate from the EHR during development and testing to allow researchers to gather rich feedback, iterate on message design, and validate functionality in a controlled setting to ensure that only vetted, effective approaches reach the live clinical environment.

A sandbox is a controlled, nonproduction environment that allows safe testing, prompt iteration, and evaluation of AI outputs without impacting live EHR systems or patient care. A preclinical sandbox can enable secure experiments with real messages, which is particularly valuable given the risks and difficulties of direct experimentation inside live EHRs [12]. A well-designed sandbox could separate front-end interactions from back-end processing, provide robust deidentification, allow plug-and-play AI components, and offer transparent telemetry estimates, including latency, token counts, and error traces. Above all, it should keep experimentation out of production until teams understand behavior, constraints, and risks [13]. Building on this need, we present a technical feasibility report describing a user interface (UI)–based sandbox developed within a secure, governed research environment.

## Technical Architecture and Development

### Setting and Governance

All development and experiments occurred within a secure research environment following institutional review board (IRB) approval and access controls. No production EHR systems were touched. The environment was equipped with an NVIDIA GRID T4-1Q graphics processing unit (GPU) to accelerate local natural language processing (NLP) pipelines and baseline model experiments.

### Software Stack and Architecture

The system is implemented in Python (version 3.12; Python Software Foundation) and Streamlit (version 1.46.1) to design the web interface. The backend is modular, with a data layer for loading and validating input, a privacy layer for deidentification, and an AI layer for task execution. Core packages included pandas and numpy for data handling, spaCy and scispaCy for entity recognition [14,15], Faker for personal health information (PHI) replacement tokens, and the OpenAI Python software development kit (SDK) configured for Azure OpenAI (GPT-4) for large language model (LLM) tasks. The deployed model was *gpt-4.1,* version *2025-04-14*, accessed using API version *2024-12-01-preview*. All dependencies were pinned in a requirements file to support reproducibility. The UI and backend are decoupled, which makes it easy to update prompts, switch model end points, or add tasks without reworking the interface. To support reproducibility, the companion source code, pinned requirements file, and synthetic test data are available in the public repository described in the Data Availability section.

### Data and Cohort Construction

We started with an IRB-approved corpus of patient portal messages over several years. For early feasibility experiments, we focused on the medical advice request message type. After standard cleaning, the working set included 6941 messages from 497 unique patient accounts in a cohort of older adults, described elsewhere [16]. This subset was chosen to capture a range of clinical questions and caregiver-authored notes, but it is not intended to represent all portal message types [16,17].

### Deidentification Pipeline

To protect patient privacy, we built a deidentification pipeline that detects and transforms personal health identifiers. Deidentification was used as an additional safeguard and as a testable privacy-preserving component, not as the only privacy control. The UI stayed within the institutional firewall, with messages only accessible by the IRB-approved study team. The pipeline uses spaCy and scispaCy named entity recognition (NER) models and custom regex patterns to identify names, health care provider titles, dates, phone numbers, addresses, and medication mentions. Replacements are deterministic per document to preserve within-message coherence; for example, the same name is mapped to the same synthetic token throughout a message. Deterministic replacement was used to preserve coherence within a single message, but synthetic placeholders were not intended to serve as persistent cross-thread pseudonyms. Conversation reconstruction was handled in the secure UI layer using internal message metadata. This design prioritizes message-level interpretability while reducing the risk that repeated placeholders could be used as linkage keys across messages. Covering medications required biomedical entity models, which we supplemented with curated lists where model coverage was limited. We sought to ensure that the output would preserve clinical semantics while removing identifiers.

### AI Tasks and Prompting Strategy

The platform was developed to enable experimentation with different AI use cases relevant to patient portal messaging. We studied four representative tasks: (1) authorship identification, to classify message authorship among 3 categories (patient, care partner, or ambiguous); (2) message categorization, to separate clinical content from administrative requests; (3) criticality flagging, to highlight urgent versus routine communications; and (4) response generation, to create editable draft replies that clinicians can review and refine.

**Figure 1.**
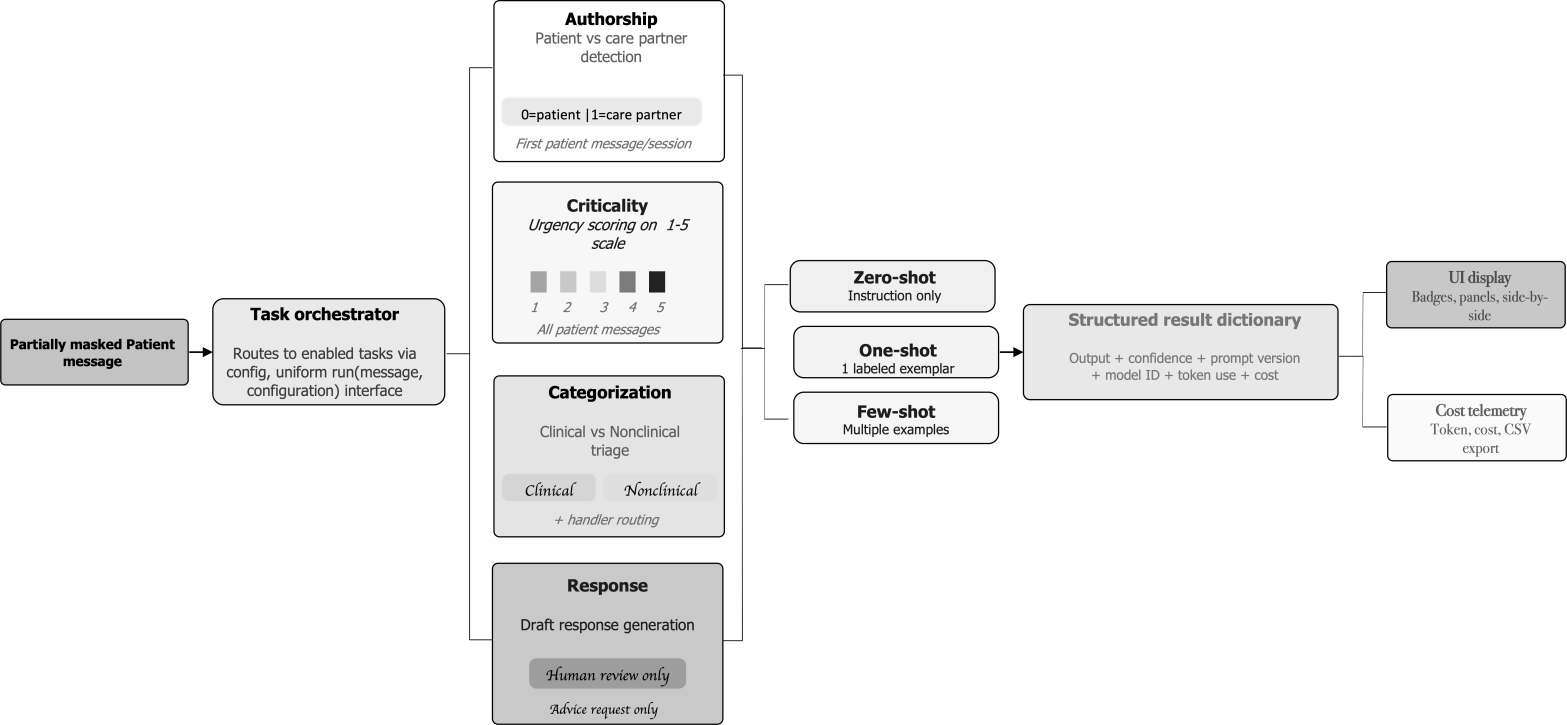
Prompt templates. UI: user interface.

All of these use cases were implemented using LLMs, tested with zero-shot, one-shot, and few-shot prompting strategies (Multimedia Appendix 1). In the one-shot setting, the model is given a single example before the target message, while in the few-shot setting, multiple examples are provided to help the model generalize the intended pattern [18]. Outputs were always framed as drafts for human review, as is currently standard practice in the use of LLM-generated drafts of patient portal messages [5]. Figure 1 summarizes the AI task orchestration layer, showing how a patient message is routed through selected use cases, processed with zero-shot, one-shot, or few-shot prompting, and returned as structured outputs for UI display and cost telemetry.

### UI and Workflows

The UI was designed to have several complementary workflows within a single platform. The most basic workflow enables the creation of scenarios from the full dataset of 6941 messages. Once loaded, the deidentification engine is automatically applied. Users can then select an enterprise identifier, which links patient and clinician messages, to reconstruct conversations. This enables side-by-side comparison of a conventional scenario with its AI-augmented counterpart, in which suggested responses or flagged messages are surfaced for clinician review. This feature supports task-level testing and the evaluation of AI within the broader flow of communication.

A second workflow allows users to enter a single message manually or upload a batch file of messages. In single-message mode, users paste a deidentified message, select one or more tasks, and view results in a side-by-side panel with the original redacted text. In batch mode, users upload a CSV file of messages; the system runs the deidentification pipeline and then executes selected tasks, producing a downloadable results file.

Additional features include a criticality ordering tool, which prioritizes messages according to urgency, and a cost analysis panel, which aggregates token use and estimates operational expenses. Together, these workflows extend the platform beyond isolated message-level testing, making it possible to simulate how AI would function in real-world messaging streams.

### Experiment Setup and Evaluation Approach

Experiments were designed to validate end-to-end functionality rather than establish clinical performance. We executed the deidentification pipeline across the 6941-message subset, then ran exemplar AI tasks in single-message and batch modes to confirm that the system produced interpretable outputs, maintained deidentification, and recorded provenance (prompt choice and task configuration).

Informal expert walkthroughs with clinical stakeholders were conducted to identify usability issues and assess whether outputs were appropriately constrained and reviewable. Participants included 2 physicians in health system information technology leadership roles, 3 internal medicine physicians, 1 nurse who triages patient portal messages, and a coalition of care partners, patient advocates, and health information technology researchers. Each walkthrough lasted 30 to 60 minutes and included an interface review, a demonstration of core features, and time for questions and feedback. We received consistent positive feedback regarding how valuable it would be to extend access to this sandbox for various AI-driven use cases. We also received feedback that more information regarding the patient’s context should be uploaded into the UI to enable users to assess the outputs. A primary usability challenge identified was that a basic level of comfort with Python was required to open the UI in the secure environment. We also incorporated insights from interviews with individuals with dementia and their care partners regarding their perceptions of AI-generated patient portal message responses [17].

We instrumented the UI to display token counts and approximate request costs. Given the volatility of LLM pricing, the cost panel was used primarily to compare prompt strategies and model choices rather than predict exact operational spending. As the focus was on the platform itself, we did not conduct head-to-head accuracy studies, which are reserved for future validation.

## System Performance and Illustrative Findings

The system executed the complete pipeline, from message ingestion to output presentation, without accessing live EHR systems. Across the 6941-message feasibility corpus, the interface supported single-message testing, batch processing, prompt selection, task execution, cost telemetry, and export of results. Because this manuscript reports technical feasibility rather than formal model evaluation, we do not present task-level output distributions or performance metrics.

AI-supported task execution was averaged approximately 5 seconds end-to-end (deidentification plus all 4 AI tasks) per message. The application included retry handling for API rate-limit errors, with up to 3 retry attempts for HTTP 429 responses. Average message length was approximately 150 to 200 words, with prompt-based processing estimated at 300 to 400 input tokens and 100 to 150 output tokens per message. Using conservative pricing assumptions, this corresponded to an estimated upper-end cost of approximately US $0.044 per message. These values are presented as operational feasibility estimates rather than production benchmarks.

We ran deidentification by replacing personal names, dates, and contact details with deterministic placeholders while preserving contextual meaning. Biomedical entity coverage for medication mentions was achieved by combining scispaCy models with a small, curated lexicon. Inspection of deidentified text confirmed that messages remained coherent and suitable for downstream analysis, although occasional overmasking of medication names was observed. To evaluate the NER pipeline used for deidentification, we tested performance on a message set. Manual review of pipeline output was conducted to identify false positives and false negatives, yielding 110 adjudicated entities. The pipeline achieved 82.1% precision, 80.9% accuracy, 95.1% sensitivity, and 39.3% specificity, with 4 false negatives identified. Because the deidentification did not achieve 100% sensitivity, all nonsynthetic messages were treated as containing PHI, including after they underwent deidentification.

In the use cases, we found that authorship prompts produced rational, auditable rationales (eg, references to third-person phrasing or caregiver self-identification) useful for reviewer feedback. We observed failure modes, such as the AI wrongly detecting care partner message authorship when in fact a patient authored the message and mentioned a spouse or family member. We also tested whether categorization and criticality prompts yielded stable labels on representative samples, and whether disagreements across prompting strategies were visible in the UI. Showing the output from the categorization and criticality prompts allowed us to collect feedback from key partners (informatics leads and physicians) that may have been otherwise hard to collect. Similarly, we were able to explore whether response drafting generated concise, editable text. We observed that the model occasionally produced overly generic and lengthy replies that did not directly address the specific clinical concern raised in the message. Batch runs were completed on the 6941-message subset and exported to a results table that combined input metadata, task outputs, and prompt provenance.

The token panel surfaced per-message and aggregate token counts, allowing users to see how different prompt templates affect inference cost. This made trade-offs between few-shot accuracy and budget more tangible during experimentation.

## Discussion

A core challenge for AI in patient portal messaging is that most tools reach clinicians long before they have been studied with realistic data and users [5,19]. Our approach prioritizes a preclinical testbed that allows health system teams to probe behavior, iterate prompts, and examine costs in a controlled setting. Several design choices were important. First, we enforced a privacy boundary: all messages were partially masked before any model access and treated as containing PHI throughout the entire process. Second, we made prompts first-class, versioned objects so that results are reproducible and modifiable. Third, we exposed batch processing alongside single-message testing to support both qualitative review and early quantitative exploration without implying clinical readiness. Our initial corpus was intentionally narrow (dementia-relevant medical advice requests from older adults), which constrains claims about performance across other message types and populations. The UI architecture, however, was built to be extensible: additional message datasets, specialized lexicons, and prompt templates can be slotted in without changing the UI.

The sandbox helps align stakeholders around evidence rather than assumptions by making experimental outputs visible and auditable. Engineers can use the environment to compare prompt templates, routing logic, and cost trade-offs; clinicians can preview which outputs reduce workload and which create new review burden; and governance teams can assess safety and equity risks before any integration decisions. The current implementation uses the OpenAI Python SDK configured for Azure OpenAI within the institutional environment. The AI layer was designed for extensibility, meaning future versions could evaluate baseline classifiers, alternative model end points, or local open-weight models as institutional policies and infrastructure evolve. However, this feasibility study did not demonstrate model substitution or formal head-to-head model comparisons. This manuscript reports a technical feasibility study rather than a formal evaluation. Future work will use this environment to conduct formal validation, comparisons with baseline classifiers and local open-weight models, turnaround times, clinician workflow impact, and staff satisfaction.

## Limitations

This work has limitations. We did not conduct a formal accuracy study or randomized usability trial; our evaluation focused on feasibility, safety controls, and instrumentation. Deidentification quality, while strong in bench tests, requires formal validation against gold standards. While the sensitivity was strong, we continued to treat the messages as containing PHI after the deidentification. The UI was developed and tested in an environment within the institution’s firewall, and nonsynthetic messages were only accessed by IRB-approved team members.

Deidentification was optimized for sensitivity to minimize PHI disclosure risk, accepting lower specificity as a deliberate trade-off. This level of overmasking, in which non-PHI terms, including some medication names, were redacted, may have reduced the semantic richness available to the LLM during preproduction testing and could have affected the quality of generated responses. The extent of this impact was not formally evaluated and represents a limitation of the current study. Medication and identifier coverage depends on biomedical NER supplemented by regex-based detection, curated medication lists, text normalization, and fuzzy matching. Although these steps improve detection of common variants and misspellings, broader use across clinical settings will require periodic updates for new medications, local abbreviations, and institution-specific terminology. The pipeline was designed with HIPAA (Health Insurance Portability and Accountability Act) Safe Harbor identifier categories in mind, but it should not be interpreted as a certified Safe Harbor or Expert Determination implementation. In settings where data would leave the institutional security perimeter, formal validation of deidentification accuracy should be evaluated before using the workflow outside a similarly governed environment.

The UI also lacked automated safeguards, such as confidence thresholds or contradiction detection. Future work will require automated safeguards and explicit strategies for long-thread management, such as truncation, summarization, or controlled context-window selection, for use cases that require reasoning across full conversation histories. Token-based cost views are approximate and will shift with pricing and prompt designs. Finally, because the system deliberately avoids live EHR integration, we did not assess downstream operational effects such as turnaround times or staff satisfaction.

The UI does not yet allow the import of demographic data; thus, we were unable to assess differential performance across demographics. With the added feature of demographic data imports in future versions, this environment could enable assessment of differential performance across demographic characteristics, such as age, race, and ethnicity, to evaluate the presence of bias and equity considerations before deployment.

Despite these limitations, a secure UI-based sandbox is a pragmatic step toward responsible AI for patient messaging. It allows organizations to study real behaviors on real messages securely, identify failure modes early, and decide which use cases, if any, merit the cost and complexity of a full integration.

## Conclusions

AI for patient portal messaging is advancing quickly, but its real impact on clinicians, patients, and care partners remains uncertain [20-22]. A secure sandbox with an accessible UI provides a practical way to test and refine AI use cases before they reach clinical workflows. Our implementation demonstrates end-to-end feasibility across the full pipeline: data ingestion, privacy preservation, configurable prompting, batch analysis, and transparent cost telemetry, all within a governed environment. The goal is not to assume benefit but to create the conditions under which benefit, risk, and fit can be studied with rigor.

## Supplementary material

10.2196/83216 Multimedia Appendix 1 Prompt templates.

## Abbreviations

EHR: electronic health record
GPU: graphics processing unit
HIPAA: Health Insurance Portability and Accountability Act
IRB: institutional review board
LLM: large language model
NER: named entity recognition
NLP: natural language processing
PHI: personal health information
SDK: software development kit
UI: user interface
