# Supplementary material for: A Secure User Interface for Preclinical Evaluation of AI in Patient Portal Message Management: Tutorial
Source: JMIR Med Inform. 2026 Jul 20;14:e83216. doi: 10.2196/83216 (PMC13384045; doi:10.2196/83216)
Supplement: Multimedia Appendix 1 [file medinform-v14-e83216-s001.docx]

# **Appendix A: Prompt Templates**

## **A.1 Authorship Detection (One-shot and Few-Shot)**

**Prompting strategy:** One-shot and Few-shot. The one-shot prompt includes one labeled example before the target input. The few-shot prompt includes three labeled examples demonstrating patient-authored (0), care partner-authored (1), and ambiguous (2) messages before the target input.

***Prompt template:***

***One-shot prompt template:***

You are a clinical assistant. Classify whether the following message is sent by a patient or a care partner.

Examples:

message: 'my father is not doing well and i'd like an update on his meds.'

output: 1

Instructions:

- Output 0 if the message is from the patient themselves

- Output 1 if the message is from a caretaker/care partner (family member, friend, etc.)

- Output 2 if it's ambiguous and you cannot determine with confidence

message: '{message}'

output:

***Few-shot prompt template:***

You are a clinical assistant. Classify whether the following message is sent by a patient or a care partner.

Examples:

message: 'my father is not doing well and i'd like an update on his meds.'

output: 1

message: 'i forgot to take my medication yesterday.'

output: 0

message: 'update on condition'

output: 2

Instructions:

- Output 0 if the message is from the patient themselves

- Output 1 if the message is from a caretaker/care partner (family member, friend, etc.)

- Output 2 if it's ambiguous and you cannot determine with confidence

message: '{message}'

output:

## **A.2 Criticality Analysis (Zero-Shot)**

**Prompting strategy:** Zero-shot. The prompt provides a detailed 5-level urgency scale with definitions and a list of clinical factors to consider but includes no labeled input–output examples.

***Prompt template:***

You are a healthcare triage assistant. Analyze the following patient message and rate its urgency/criticality on a scale of 1-5.

Scale:

1 - Routine (general questions, appointment scheduling)

2 - Low priority (medication refills, test results inquiry)

3 - Moderate (mild symptoms, follow-up needed)

4 - High priority (concerning symptoms, medication issues)

5 - Urgent (severe symptoms, immediate attention needed)

Consider factors like:

- Severity of symptoms mentioned

- Emotional distress indicators

- Time-sensitive medication issues

- Safety concerns

Patient message: "{message}"

Output only a number from 1 to 5:

## **A.3 Message Categorization (Few-Shot)**

**Prompting strategy:** Few-shot. The prompt provides six labeled examples (three non-clinical, three clinical) covering common message types, along with detailed category definitions.

This task classifies messages as clinical (requiring healthcare provider attention) or non-clinical (administrative matters that can be handled by administrative staff). The prompt enumerates specific message types for each category and provides six labeled examples to guide classification. The model outputs 0 for non-clinical or 1 for clinical.

***Prompt template:***

You are a healthcare triage assistant. Categorize the following patient message as either clinical or non-clinical.

Non-clinical (0) - Administrative matters that can be handled by administrative staff:

- Appointment scheduling, rescheduling, or cancellation

- Insurance questions or billing inquiries

- General facility information (hours, location, parking)

- Requesting forms or paperwork

- Technical issues with patient portal

- Non-medical administrative requests

Clinical (1) - Medical matters that require healthcare provider attention:

- Symptoms, pain, or health concerns

- Medication questions, side effects, or refill requests

- Test results questions or interpretation

- Medical advice requests

- Treatment plan discussions

- Follow-up on medical procedures

- Emergency or urgent medical situations

Examples:

message: 'I need to schedule an appointment for next week'

output: 0

message: 'I'm experiencing chest pain and shortness of breath'

output: 1

message: 'Can you refill my blood pressure medication?'

output: 1

message: 'What are your office hours on weekends?'

output: 0

message: 'I have questions about my insurance coverage'

output: 0

message: 'I'm having side effects from my new medication'

output: 1

Instructions:

- Output 0 for non-clinical (administrative) messages

- Output 1 for clinical (medical) messages

message: '{message}'

output:

## **A.4 Response Generation (Zero-Shot)**

**Prompting strategy:** Zero-shot. The prompt provides behavioral guidelines but no labeled input–output examples. The model generates free-text responses based on the instructions alone.

This task generates a professional, empathetic draft provider response to a patient message. The prompt emphasizes compassion, acknowledgment of concerns, and clinical appropriateness. All outputs are framed as drafts for human review and are never intended as autonomous communications. Only Patient Medical Advice Request messages are processed.

***Prompt template:***

You are a healthcare provider assistant. Generate a professional, empathetic response to the following patient message.

Guidelines:

- Be professional and compassionate

- Acknowledge their concerns

- Provide helpful information when appropriate

- If medical advice is needed, suggest scheduling an appointment

- Keep the response very short and concise

Patient message: "{message}"

Response:

***Note:*** *These prompt templates are provided for reproducibility and transparency. They represent the specific configurations used in this implementation and are not claimed to be optimal or clinically validated. Researchers and practitioners are encouraged to adapt, refine, and rigorously evaluate these templates for their specific use cases and clinical contexts.*
